# The GPR30 Receptor Is Involved in IL-6-Induced Metastatic Properties of MCF-7 Luminal Breast Cancer Cells

**DOI:** 10.3390/ijms25168988

**Published:** 2024-08-18

**Authors:** Ana Carolina Tirado-Garibay, Betzabe Ruiz-Barcenas, Julia Isabel Rescala-Ponce de León, Alejandra Ochoa-Zarzosa, Joel E. López-Meza

**Affiliations:** Centro Multidisciplinario de Estudios en Biotecnología, Facultad de Medicina Veterinaria y Zootecnia, Universidad Michoacana de San Nicolás de Hidalgo, Km 9.5 Carretera Morelia-Zinapécuaro, Posta Veterinaria, Morelia 58893, Michoacán, Mexico; 1050629h@umich.mx (A.C.T.-G.); l21121038@morelia.tecnm.mx (B.R.-B.); l21121031@morelia.tecnm.mx (J.I.R.-P.d.L.); alejandra.ochoa@umich.mx (A.O.-Z.)

**Keywords:** GPR30, IL-6, epithelium-mesenchymal transition, luminal, metastasis

## Abstract

Luminal breast cancer has a high incidence worldwide and poses a severe health threat. Estrogen receptor alpha (ER-α) is activated by 17β-estradiol (E2), and its overexpression promotes cancerous characteristics. Luminal breast cancer is an epithelial type; however, the cytokine IL-6, secreted by cells within the tumor microenvironment, stimulates the epithelial-to-mesenchymal transition (EMT) and promotes metastasis. Also, IL-6 decreases ER-α levels, favoring the tamoxifen (TMX) resistance development. However, genes under E2 regulation continue to be expressed even though this receptor is absent. GPR30 is an alternative E2 receptor present in both luminal and aggressive triple-negative breast cancer and is related to TMX resistance and cancer progression. The roles of GPR30 and IL-6 in metastasis have been individually established; however, their interplay remains unexplored. This study aims to elucidate the role of GPR30 in IL-6-induced metastatic properties of MCF-7 luminal breast cancer cells. Results showed that GPR30 contributes to the E2-induced MCF-7 proliferation because its inhibition with the antagonist G15 and the *Pertussis* toxin (PTX) reduced it. Besides, GPR30 upregulated vimentin and downregulated E-cadherin levels in MCF-7 and TMX-resistant (R-TMX) cells and is also involved in the IL-6-induced migration, invasion, and TMX resistance in MCF-7 cells. In addition, in MDA-MB-231 triple-negative cells, both basal and IL-6-induced metastatic properties were related to GPR30 activity. These results indicate that the GPR30 receptor regulates the EMT induced by IL-6 in breast cancer cells.

## 1. Introduction

Breast cancer is a rising health problem worldwide; this neoplasia originates in the mammary gland and then develops metastasis. Breast cancer can be classified upon the over-expression of three receptors: the estrogen receptor alpha (ER-α), the progesterone receptor (PR), and the human epidermal growth factor receptor 2 (HER2). The luminal A subtype is characterized by the over-expression of ER-α responding to 17β-estradiol (E2), promoting proliferation; it presents a high incidence worldwide and has reduced aggressiveness due to its epithelial phenotype [1,2]. On the contrary, the triple-negative subtype lacks the three receptor over-expression and has a lower incidence than the luminal type; however, it presents a higher aggressiveness due to its mesenchymal phenotype [3]. The characteristics of the luminal subtype have allowed the development of therapies against specific targets such as ER-α [4]; however, the acquisition of resistance to drugs such as tamoxifen (TMX, which binds to ER-a) [5,6] and the development of metastatic properties such as migration and invasion have been reported [7].

The tumor microenvironment plays a crucial role in cancer development because it modifies the properties of cancer cells; this is integrated by immune and connective cells that secrete cytokines, such as IL-6. This cytokine induces the EMT, favoring metastasis development in luminal breast cancer cells [7,8]. Also, IL-6 has been related to TMX resistance through a decrease in ER-α levels [9,10]; however, the cancer cells continue expressing genes under E2 regulation [11], suggesting the participation of another receptor for this hormone. GPR30/GPER is an alternative E2 receptor that can regulate the expression of ER-α target genes [12]. This receptor is expressed in luminal and triple-negative breast cancer cells [13] and has been related to TMX resistance [14] and cancer progression [15,16]. GPR30 and IL-6 have been involved in metastasis, but an interplay between them has not been reported.

The metastasis is the main difficulty of the present therapy against cancer because the dispersion and heterogeneity of affected cells complicate their elimination [17]. Despite the successful treatment for luminal cancer, about 40% of the patients develop resistance to the drugs, and cancer becomes more aggressive [18]; for this reason, it is important to understand the mechanisms through which the luminal breast cancer cells develop metastatic proprieties to find potential targets and strategies to control and eradicate this pathology. This work aims to determine the GPR30 role in the metastatic properties induced by IL-6 in luminal breast cancer cells.

## 2. Results

### 2.1. GPR30 Regulates the Proliferative Effects of E2 and IL-6 in Breast Cancer Cells

**Figure 1:**
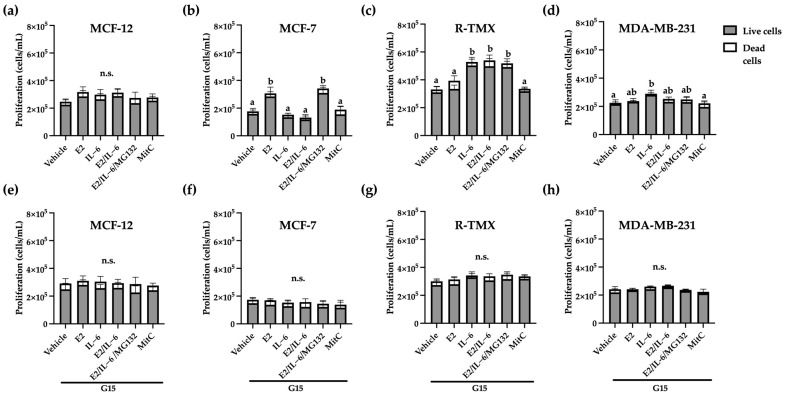
The GPR30 antagonist G15 inhibits the proliferation of breast cancer cells induced by E2 and IL-6. (**a**,**e**) MCF-12, (**b**,**f**) MCF-7, and (**c**,**g**) R-TMX cell proliferation was evaluated at 24 h. MDA-MB-231 (**d**,**h**) cell proliferation was evaluated at 48 h. Data were obtained by trypan blue exclusion assay in the presence of E2 (10 nM), IL-6 (50 ng/mL), MG132 (0.03 μM), and G15 (0.625 μM). Each bar shows the mean of triplicates ± SE of three independent experiments. Different letters denote significant differences within the treatments (one-way ANOVA and Tukey’s comparison, *p* ≤ 0.05). n.s. = not significant.

One of the initial hallmarks of cancer is sustained proliferation, which is responsible for the neoplasia generation [19]. Based on this, we evaluated the effect of E2 and IL-6 on breast cancer cell proliferation by trypan blue exclusion assay and mitomycin C (Mit C) as an antiproliferative control. The proliferation of MCF-12 (cells from healthy mammary glands that do not overexpress receptors such as ER-α) was not affected by E2 nor IL-6 (Figure 1a). Also, MCF-7 cells stimulated their proliferation in the presence of E2, which was inhibited by IL-6, a fact that was expected by the evidence of proteasomal regulation of E2 signaling by the cytokine [10]; in agreement, this effect was reverted by the proteasomal inhibitor MG132 (Figure 1b). Besides, the proliferation of MCF-7 tamoxifen-resistant (R-TMX) was induced only by IL-6 and was independent of E2 (Figure 1c). As expected, the proliferation of triple-negative breast cancer cells MDA-MB-231 was not affected by E2, but IL-6 stimulated it (Figure 1d). The GPR30 antagonist G15 (Figure 1e–h) or PTX toxin, a G-protein (αi) inhibitor (Figure S1), inhibited all the proliferative effects, suggesting the GPR30 participation in the proliferation stimulated by E2 and IL-6 in breast cancer cells.

### 2.2. IL-6 Promotes the Expression of Vimentin through GPR30

**Figure 2:**
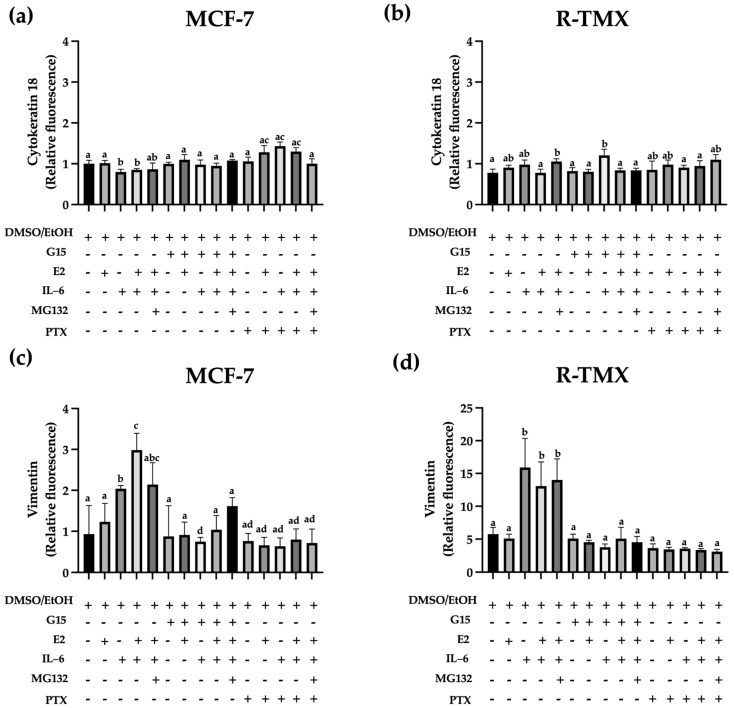
IL-6 regulates the mesenchymal protein pattern expression in MCF-7 and R-TMX cells. (**a**,**b**) Cytokeratin 18 and (**c**,**d**) vimentin expression of MCF-7 and R-TMX cells was assessed at 48 h in the presence of E2 (10 nM), IL-6 (50 ng/mL), MG132 (0.03 μM), G15 (0.625 μM), and PTX (100 ng/mL) by flow cytometry. Each bar shows the mean of triplicates ± SE of three independent experiments. Different letters denote significant differences within the treatments (one-way ANOVA and Tukey’s comparison, *p* ≤ 0.05).

Tumor formation is insufficient to classify neoplasia as cancer; metastasis development is compulsory [1]. The EMT implies the loss of cell junctions, such as desmosomes, by decreasing adherent protein and increasing motility characteristics [20]. Cytokeratin 18 is an intermediate filament expressed in epithelial carcinoma and is diminished in basal subtypes, contrary to vimentin, which is implicated in the migration mechanism [21]. We evaluate the cytokeratin 18 and vimentin expression in MCF-7 and R-TMX cells. Results showed a slight decrease in cytokeratin 18 levels in MCF-7 cells stimulated with IL-6, which was reverted when cells were treated with the GPR30 antagonist G15 or PTX (Figure 2a). An opposite behavior was observed with vimentin (Figure 2c) because it was increased (~2-fold), suggesting that IL-6 promotes EMT protein expression through GPR30. Also, vimentin levels were strongly increased in R-TMX cells by IL-6 (~3-fold) (Figure 2c,d). Interestingly, R-TMX cells showed a higher vimentin level in relation to MCF-7 cells. In both cases, these increments were reverted by G15 or PTX (Figure 2). Despite this mesenchymal behavior, cytokeratin 18 levels were similar in both cellular types (Figure 2).

### 2.3. GPR30 Regulates Basal and IL-6-Induced Migration in Breast Cancer Cells

**Figure 3:**
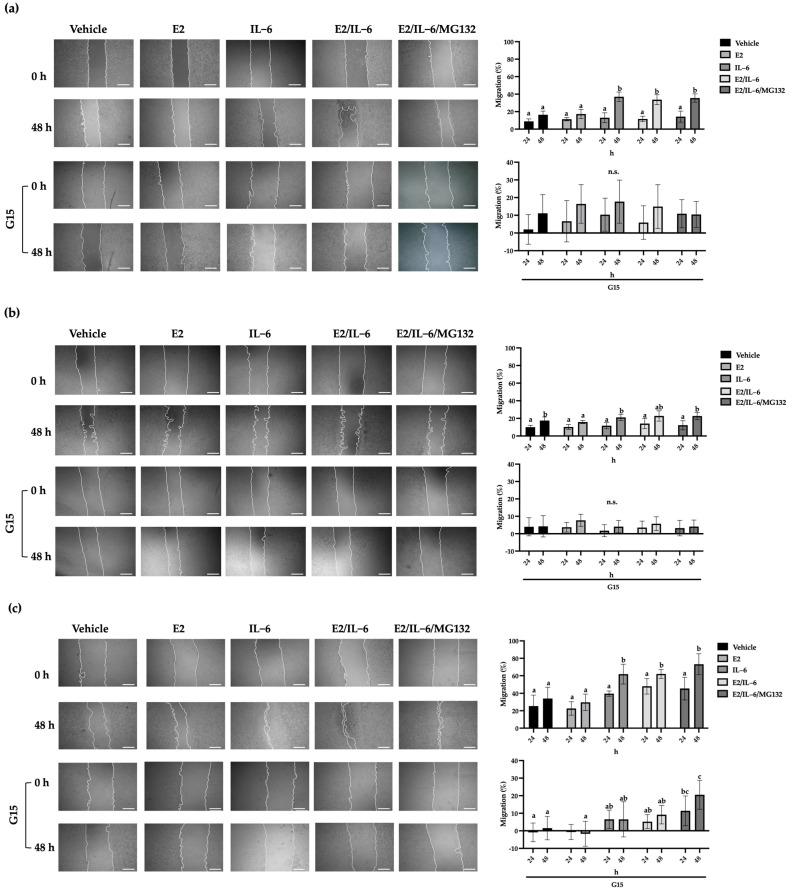
Breast cancer cell migration. (**a**) MCF-7, (**b**) R-TMX, and (**c**) MDA-MB-231 cell migration was evaluated by wound healing assay at 24 and 48 h in the presence of E2 (10 nM), IL-6 (50 ng/mL), MG132 (0.03 μM), and G15 (0.625 μM). The photographs are 4X, and the scale bar represents 500 μm. Each bar shows the mean of triplicates ± SE of three independent experiments. Different letters denote significant differences within the treatments (two-way ANOVA and Tukey’s comparison, *p* ≤ 0.05).

The increase in vimentin levels is related to a directed migration [22]. This process includes detachment and mobilization of the cells. For this, we evaluate the migratory capability of breast cancer cells by wound healing assay. MCF-12 cell migration was not affected by E2 or IL-6 (Figure S2). Also, the MCF-7 cells did not show any changes in their migration rate in response to E2 (20%); however, the IL-6 doubled the migration capability at 48 h (Figure 3a), which was inhibited by the GPR30 antagonist G15 (Figure 3a) or PTX (Figure S3). Similar behavior was observed in R-TMX cells (Figure 3b). Noteworthy, the MDA-MB-231 cell line showed a basal migration of 35%, which was doubled by IL-6 (60%) at 48 h; however, G15 inhibited both the basal and stimulated migration (Figure 3c). These results suggest that GPR30 is involved in the migration of breast cancer cells.

### 2.4. IL-6 Promotes Breast Cancer Cell Invasion through the GPR30 Receptor

**Figure 4:**
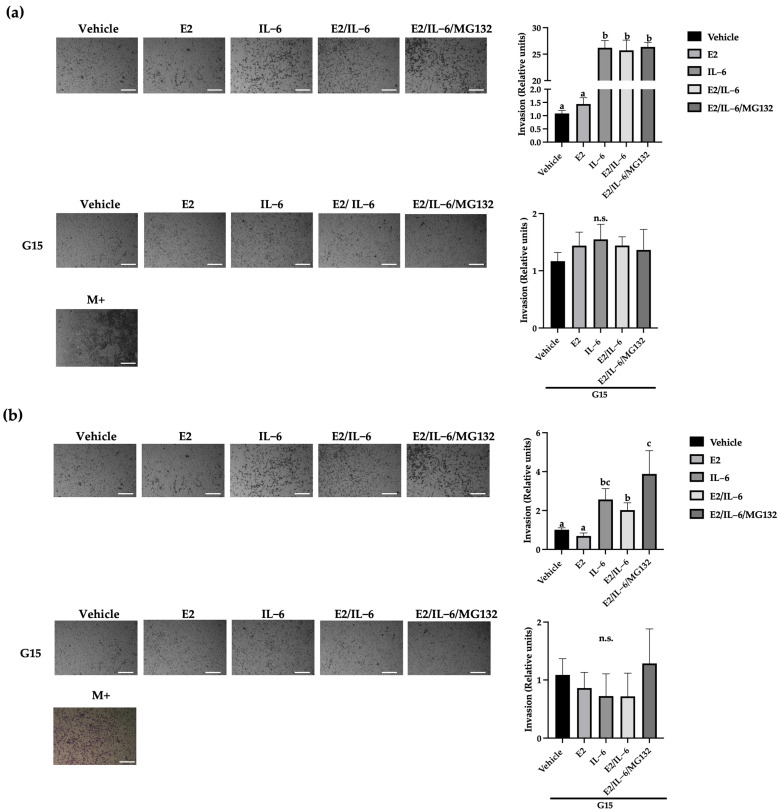

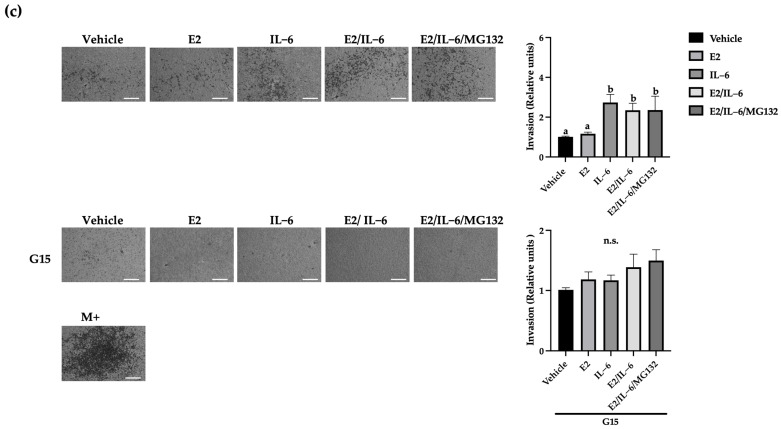
Breast cancer cell invasion. (**a**) MCF-7, (**b**) R-TMX, and (**c**) MDA-MB-231 cell invasion was assessed by Transwell chamber assay at 24 h of stimulation with E2 (10 nM), IL−6 (50 ng/mL), MG132 (0.03 μM), and G15 (0.625 μM). M+ = cells cultured in F-12 medium supplemented with FBS 10%. 10X photographs, scale bar represents 250 μm. Each bar shows the mean of triplicates ± SE of three independent experiments. Different letters denote significant differences within the treatments (one-way ANOVA and Tukey’s comparison, *p* ≤ 0.05). n.s. = not significant.

Once the malignant cells arrive at secondary organs, they invade them to develop metastatic tumors. This process involves a directed migration through the extracellular matrix [1,23]. We evaluate the IL-6-induced invasion of breast cancer cells by Transwell assay. The MCF-12 cells did not show invasiveness even though they were stimulated with E2 or IL-6 (Figure S2). Besides, the MCF-7 cells did not increase their invasion capability in response to E2 (Figure 4a). However, the MCF-7 invasiveness was induced by IL-6 (~25-fold), which was reversed by the GPR30 antagonist G15 (Figure 4a) or PTX (Figure S3). Similar results were observed in R-TMX cells, but the IL-6 induction was lower (~2-fold) (Figure 4b). Also, the invasion of MDA-MB-231 cells was enhanced by IL-6 (~3-fold) (Figure 4c); interestingly, the basal and induced invasion was inhibited by G15, highlighting the role of GPR30 in the cell invasion of breast cancer cells.

### 2.5. GPR30 Is Also Involved in the IL-6-Induced TMX Resistance in Luminal Breast Cancer

One of the main problems of current therapies against cancer is the development of resistance to the drugs. TMX resistance is the most common in breast cancer. We assessed if IL-6 induces TMX resistance in MCF-7 cells. Interestingly, cells treated with IL-6 showed an increased time-dependent TMX resistance (Figure S4, Table 1). Noteworthy, the GPR30 antagonist G15 diminished the TMX IC_50_ values in cells treated with IL-6, suggesting a re-sensibilization to TMX (Figure S4, Table 1). Also, the G15 significantly reduced the IC_50_ of TMX in R-TMX cells.

**Figure 5:**
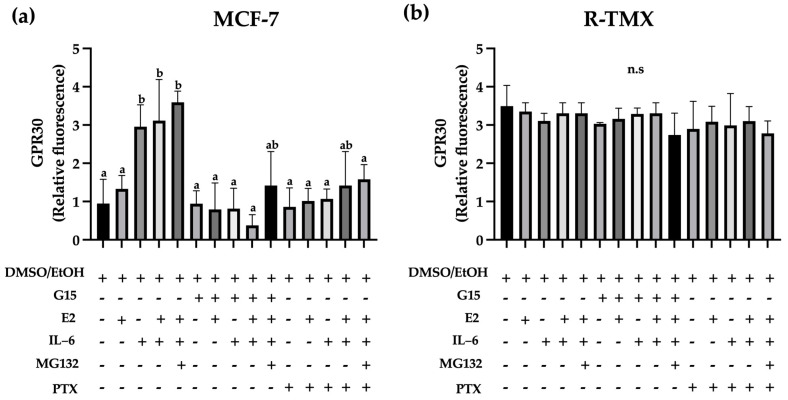
GPR30 levels in breast cancer cells. (**a**) GPR30 levels in MCF-7 cells and (**b**) GPR30 levels in R-TMX cells at 48 h in the presence of E2 (10 nM), IL-6 (50 ng/mL), MG132 (0.03 μM), G15 (0.625 μM), and PTX (100 ng/mL). Protein levels were assessed by flow cytometry as described in the Materials and Methods section. Each bar shows the mean of triplicates ± SE of three independent experiments. Different letters denote significant differences within the treatments (one-way ANOVA and Tukey’s comparison, *p* ≤ 0.05). n.s. = not significant.

The TMX resistance and metastasis development have been related to increased GPR30 levels; this receptor is located in membranous organelles and can be mobilized in cancer [14]. Based on this, we evaluated the intracellular GPR30 levels. IL-6 increased GPR30 levels (3-fold) in MCF-7 cells (Figure 5a), which was unaffected by E2. Noteworthy, the antagonist G15 or PTX reverted the IL-6 induction. Also, R-TMX cells showed increased GPR30 levels concerning MCF-7 cells; however, all treatments did not modify them (Figure 5b).

## 3. Discussion

Cancer is a disease characterized by the proliferative and metastatic capacity of the cancerous cells, and the tumor microenvironment can influence these properties. In the present work, we evaluated the effect of IL-6 cytokine on breast cancer cell proliferation, migration, and invasion properties, which are relevant for EMT. We showed evidence that GPR30, an alternative non-canonical E2 receptor, regulates the EMT induced by IL-6 in MCF-7 cells.

Luminal breast cancer is epithelial, which means that it has accentuated neoplastic properties and requires external stimulation to acquire a metastatic phenotype [24]. MCF-7 cells belong to this subtype, and their stimulation with E2 promotes their proliferation; however, IL-6 inhibits the effect of the hormone because it induces the proteasomal degradation of ER-α [25]. This report agrees with our results because the IL-6 effect on MCF-7 proliferation was reverted by the proteasomal inhibitor MG132 (Figure 1). Also, the tamoxifen-resistant variant and the triple-negative variant did not respond to the E2 stimulus (Figure 1), which was associated with a decrease in ER-α levels, as reported [26]. Besides, we observed an IL-6-induced proliferation in R-TMX cells, which has been reported in cancer cells that do not respond to estrogen; this induction could be regulated through the epidermal growth factor receptor (EGFR) [27]. It is noteworthy that both proliferative effects were inhibited by the GPR30 antagonist G15 or the *Pertussis* toxin (PTX); the last suggests that GPR30 activity is associated with an inhibitory alpha subunit (Gαi) in these models. Also, it has been reported that ER-α and GPR30 receptors may interact physically and that inhibition of one of these decreases the proliferative effect of E2 [28,29], which agrees with the results of this work. In addition, the inhibition of the effect of IL-6 by the antagonist G15 could be due to the cross-linking of the signaling pathways of EGFR and GPR30 since the latter can transactivate the former. However, further experiments are necessary to clarify it.

On the other hand, the epithelial-mesenchymal transition (EMT) is a prerequisite for metastasis development. It can be detected by a decrease in cytokeratin 18 (CTK 18) levels and an increase in vimentin (Vim) levels [30]. We showed that E2 did not affect the levels of these proteins in MCF-7 and R-TMX cells, but IL-6 induced the pattern mentioned above (Figure 2). Likewise, the antagonist G15 or PTX inhibited the IL-6 effect, allowing the cells to recover their basal levels of CTK 18 and Vim, suggesting that the GPR30 receptor is involved in the EMT induced by IL-6. In support of the above, the migration and invasion properties of MCF-7 and R-TMX cells induced by IL-6 were inhibited by the antagonist G15 or PTX (Figure 3 and Figure 4). Interestingly, in MB-MDA-231 cells, we observed a similar behavior (Figure 4). These results suggest that GPR30 has a relevant role in the EMT in the different phases of breast cancer cells, and it could be a target for future drugs.

**Figure 6:**
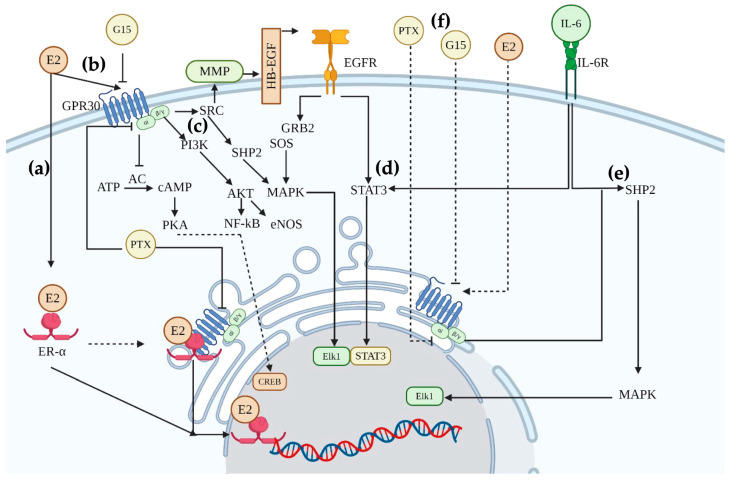
GPR30 signaling pathway. (**a**) The canonical E2 signaling through ER-α and the physical interaction of GPR30 with ER-α are represented. Also, the GPR30 activation by E2 is shown (**b**); this receptor has been associated with an inhibitory Gα-protein, which impairs the adenylate cyclase activity and, therefore, the CREB nuclear translocation. (**c**) The βγ units trigger the PI3K/AKT pathway and the transactivation of EGFR through the release of HB-EGF. These units also activate the MAPK pathway through SRC/SHP2. (**d**) The EGFR and IL-6 signaling crosstalk at the STAT3 level. (**e**) The GPR30 and IL-6 pathways converge at the SHP2 level, resulting in gene expression. (**f**) The GPR30 antagonist G15 impairs the ligand activation of GPR30, and the Pertussis toxin (PTX) inhibits the Gαi activation. Created with BioRender.com.

GPR30 is located in the endoplasmic reticulum of luminal and triple-negative breast cancer cells [31], and an increase in this receptor has been related to the aggressiveness of the pathology [31,32]. In agreement, we showed an intracellular rise in GPR30 receptor levels in MCF-7 cells induced with IL-6 (Figure 5a). Also, the intracellular levels of GPR30 in R-TMX were similar in all treatments. In this sense, TMX resistance has been related to the membrane localization of GPR30, which may favor EGFR transactivation. The latter is overactivated in drug-resistant breast cells and regulates the expression of GPR30 [32,33]. However, further studies are required to evaluate the membrane abundance of GPR30 in R-TMX cells. Besides, the localization and activity of GPR30 depend not only on the cancer stage but also on the cell type. This receptor has a pro-tumorigenic activity in breast, lung, and glioblastoma; interestingly, the latter two organs are metastatic targets of breast cancer. On the opposite, GPR30 has an anti-tumorigenic role in ovarian and pancreatic cancers, and as expected, breast cancer rarely metastases to these tissues [34,35]. In addition to the remarkable GPR30 participation in metastatic properties of cancer cells and the correlation of their levels with poor prognosis in cancer tissues [15,16], further studies of this receptor in vivo are needed to assess its role in breast cancer. In summary, the results support that the metastatic properties of MCF-7 cells regulated by IL-6 involve the participation of the receptor GPR30 (Figure 6), which could be evaluated as a target for future drugs.

## 4. Materials and Methods

### 4.1. MCF-12, MCF-7, and MDA-MB-231 Cell Culture

The breast cancer cell lines MCF-7 (cell line was acquired from ATTC, Manassas, VA, USA) and MDA-MB-231 and the healthy breast line MCF-12 (both cell lines were kindly donated by Dra. Carmen Aceves from Instituto de Neurobiología, UNAM, Juriquilla, Querétaro, México) were used in this work. MCF-12 and MCF-7 cells were cultured in F12 medium supplemented with 5% fetal bovine serum (FBS) (Biowest, Rue du Vieux Bourg, Nuaillé, France, cat. S1820-500), 5% calf serum (ST) (Biowest cat. S0400-500), 1% penicillin/streptomycin (Gibco, Waltham, MA, USA, cat. 15140122), and 0.05% amphotericin B (Sigma Aldrich, St. Louis, MO, USA, cat. A2942). The culture of MDA-MB-231 cells was carried out with DMEM supplemented with the abovementioned composition, adding L-glutamine (Gibco cat. 20530-081) to a final concentration of 200 mM. The three cell lines were cultured at 37 °C and 5% CO_2_.

### 4.2. Treatments

The cell lines were exposed to the vehicle DMSO (Sigma Aldrich cat. D1436) at 0.005% and ethanol (J.T. Baker, Monterrey, Nuevo León, México, cat. 9000-02) at 0.05% as controls. The treatments consisted of an individual or combined exposure to E2 10 nM (Sigma Aldrich E2758), IL-6 50 ng/mL (Peprotech, Cranbury, NJ, USA, 200-06), G15 0.625 μM (Peprotech cat. 1160560), MG132 0.3 μM (Sigma Aldrich C2211), and PTX 100 ng/mL (Sigma Aldrich P2980).

### 4.3. Tamoxifen Resistant Line (R-TMX)

10,000 MCF-7 cells were cultured per well in a 96-well plate and exposed to 1 μM tamoxifen (TMX) in a supplemented F12 medium in a final volume of 100 μL [34,35]. The cells were subcultured every three days, placing the same initial cell quantity and exposed to the same culture medium for 16 generations. Throughout this period, the TMX toxicity in the cells was evaluated biweekly by trypan blue exclusion, ultimately determining an IC_50_ above 20 μM, thereby considering that the cells had acquired resistance to the drug. These cells were maintained in F12 medium supplemented with FBS (5%), ST (5%), penicillin/streptomycin (1%), amphotericin B (0.05%), and TMX (1 μM) at 37 °C and 5% CO_2_.

### 4.4. Proliferation and TMX Cytotoxicity

10,000 MCF-12, MCF-7, and R-TMX, and 20,000 MDA-MB-231 cells/well were cultured in 96 well culture plates (NEST, Wuxi, Jiangsu, China, cat. 701001) in a supplemented medium (100 μL/well) for 24 h, after which they were synchronized by starvation for 18 h with F12 medium without phenol red. Subsequently, treatments were added for 24 for the first three cell types and 48 h for the triple negative, and cells were harvested with 50 μL of trypsin 0.04%; when the cells were in suspension, 50 μL of the supplemented medium was added. Cell viability was assessed by trypan blue assay in a Bio-Rad (Hercules, CA, USA) automatic cell counter (Model TC20), using cell suspension and trypan blue in 1:1 proportion. To evaluate the cytotoxicity of TMX, MCF-7 and R-TMX cells were cultured following the previously described methodology, with 48 h exposure to the following treatments: TMX (1, 2, 3, 4, 5, 10, 15, and 20 μM), IL-6 (50 ng/mL), and G15 (0.625 μM), individually and together.

### 4.5. Flow Cytometry

80,000 MCF-7 and R-TMX cells were cultured in 24-well plates, starved by 18 h, and then stimulated for 48 h with the treatments. After this time, they were harvested with trypsin 0.04%, centrifugated at 1500 rpm per 15 min at 4 °C and washed with PBS, subsequently fixed with methanol absolute for 15 min and permeabilizated with Tween 20 (0.5%) for 20 min. The antibodies were purchased from Santa Cruz Biotechnology (Dallas, TX, USA), Cell Signaling Technology (Danvers, MA, USA), and Abcam (Trumpington, Cambridge, UK). The cells were incubated with the following antibodies: 1:500 anti-vimentin (SC-6260), anti-cytokeratin 18 (SC-32329), and 1:1000 anti-GPR30 (ab39742) overnight at 4 °C. Next, the cells were centrifugated as described above and then incubated with anti-mouse IgG Fab2 Alexa Fluor 488 (4408S) or anti-rabbit IgG Fab2 PE (8885S) 1:500 for 2 h at 4 °C in the dark and analyzed in a BD Accuri™ C6 cytometer (BD Biosciences, NJ, USA) set to 10,000 events per assay. The data were analyzed in FlowJo Software v10 and were normalized concerning the vehicle.

### 4.6. Wound Healing Assay

Cell migration was assessed by wound healing assay. 80,000 cells were cultured per well in 24-well plates (NEST cat. 702001) for 24 h and starved by 18 h, and proliferation was inhibited with mitomycin C (4 μg/mL, Sigma Aldrich cat. M0503) for 2 h. Then, the wounds were made in a cross shape with a 200 μL tip and a sterile slide. Two washes were then performed with PBS, and the treatments were placed in a final volume of 400 μL per well. Images of the wounds were captured at times of 0, 24, and 48 h at 4X using an inverted microscope. The images were analyzed using ImageJ software v1.8.0; the wound area was measured with the wound healing tool expansion, using the parameters variance 3, threshold 250, and ratio 0. Four photographs per treatment per time point were analyzed for each assay, and this was performed three times.

### 4.7. Evaluation of Cell Invasion by Transwell Chamber

Cell cultures were carried out in 50 mm diameter dishes at confluence; subsequently, they were synchronized by starvation for 18 h. Simultaneously, 100 μL of matrigel (1.25 mg/mL) was placed in the inserts of 8 μm Transwell plates (Corning incorporated-Costar^®^, Glendale, CA, USA, cat. 3422) and incubated at 37 °C for 24 h. After this time, the cells were harvested, and 25,000 cells were placed in a volume of 20 μL on the polymerized matrigel. The corresponding treatments were placed in the 24-well plate in a volume of 400 μL per well, and the Transwell inserts were placed on top of them. They were incubated at 37 °C and 5% CO_2_ for 24 h. After this, the matrigel was removed, and the insert was washed twice with PBS, which was repeated after each subsequent process. The cells were then fixed with 4% p-formaldehyde, permeabilized with methanol (J. Baker cat. 9070-03), and finally, dyed with crystal violet (Sigma Aldrich cat. C0775) 0.04%. The images were captured on the inverted microscope (Zeiss Primovert) with the 4X and 10X objectives and analyzed with ImageJ software v1.8.0.

### 4.8. Statistic Analysis

Statistical analyses of the triplicated assays were performed in Prisma Graph Pad software v8 with group testing using two-way ANOVA and post hoc Tukey (α ≤ 0.05) for migration evaluation and one-way ANOVA for proliferation, flow cytometry, and invasion assays. The TMX resistance was analyzed by *t*-student *p* ≤ 0.05.

## 5. Conclusions

The results indicate that the GPR30 receptor is involved in cell proliferation induced by IL-6 and E2 in breast cancer and IL-6-induced metastatic properties, such as migration and invasion in luminal, TMX-resistant, and triple-negative cancer cells. The GPR30 antagonist G15 and the Gα inhibitor PTX reduced these properties, supporting the GPR30 role in the metastatic phenotype induced by IL-6 in breast cancer.

## Figures and Tables

**Table 1:** Mean lethal concentrations (IC50) of TMX in MCF-7 cells stimulated with IL−6 (50 ng/mL) and G15 (0.625 μM).

Cell Conditions	IC_50_ TMX (μM)
MCF-7	3.8
MCF-7 + G15	4.26
MCF-7 + IL-6 (24 h)	10.98 *
MCF-7 + IL-6 (48 h)	12.69 *
MCF-7 + IL-6 (24 h) + G15	3.87
MCF-7 + IL-6 (48 h) + G15	5.08 *
R-TMX	>20 *
R-TMX + G15	9.05 *

Data shows the mean of triplicates ± SE of three independent experiments. * represents a statistically significant difference in comparison to MCF-7 cells (*t*-student, *p* ≤ 0.05).

## Data Availability

The raw data supporting the conclusions of this article will be made available by the authors, without undue reservation, to any qualified researcher.

## Supplementary Materials

The supporting information can be downloaded at https://www.mdpi.com/article/10.3390/ijms25168988/s1.
