# Supplementary material for: The GPR30 Receptor Is Involved in IL-6-Induced Metastatic Properties of MCF-7 Luminal Breast Cancer Cells
Source: Int J Mol Sci. 2024 Aug 18;25(16):8988. doi: 10.3390/ijms25168988 (PMC11354767; doi:10.3390/ijms25168988)
Supplement: Supplementary file 1 [file ijms-25-08988-s001.zip › ijms-3146296-supplementary.pdf]

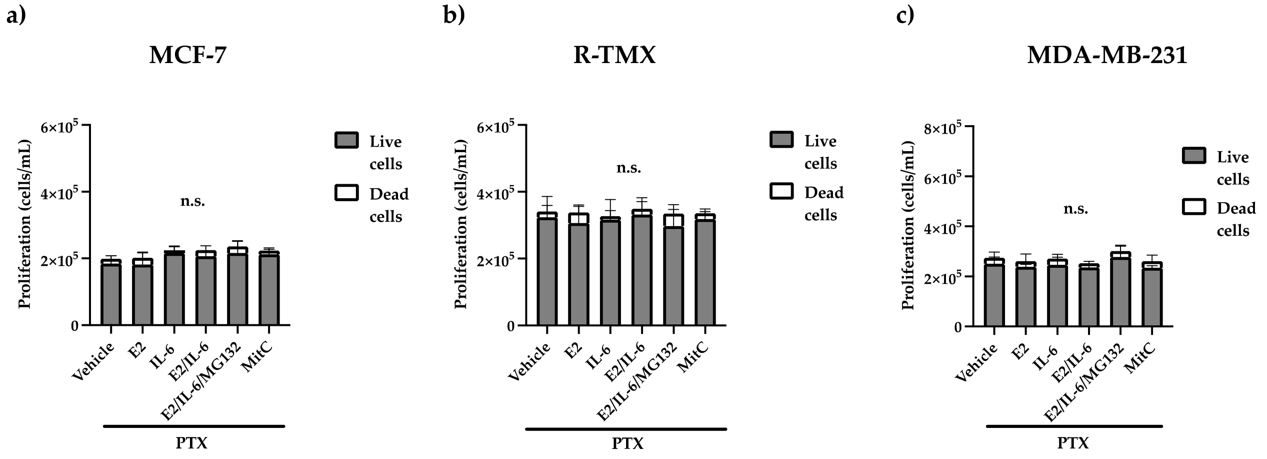

Figure S1. The PTX inhibits the proliferation of breast cancer cells induced by E2 and IL-6. (**a**) MCF-7, (**b**) R-TMX and (**c**) MDA-MB-231 cell proliferation was evaluated at 24 h. Data were obtained by trypan blue exclusion assay. E2 (10 nM), IL-6 (50 ng/mL), MG132 (0.03 µM), and PTX (100 ng/mL). Each bar shows the mean of triplicates ± SE of three independent experiments. One-way ANOVA, post hoc Tukey, $p \leq 0.05$. n.s.= not significant.

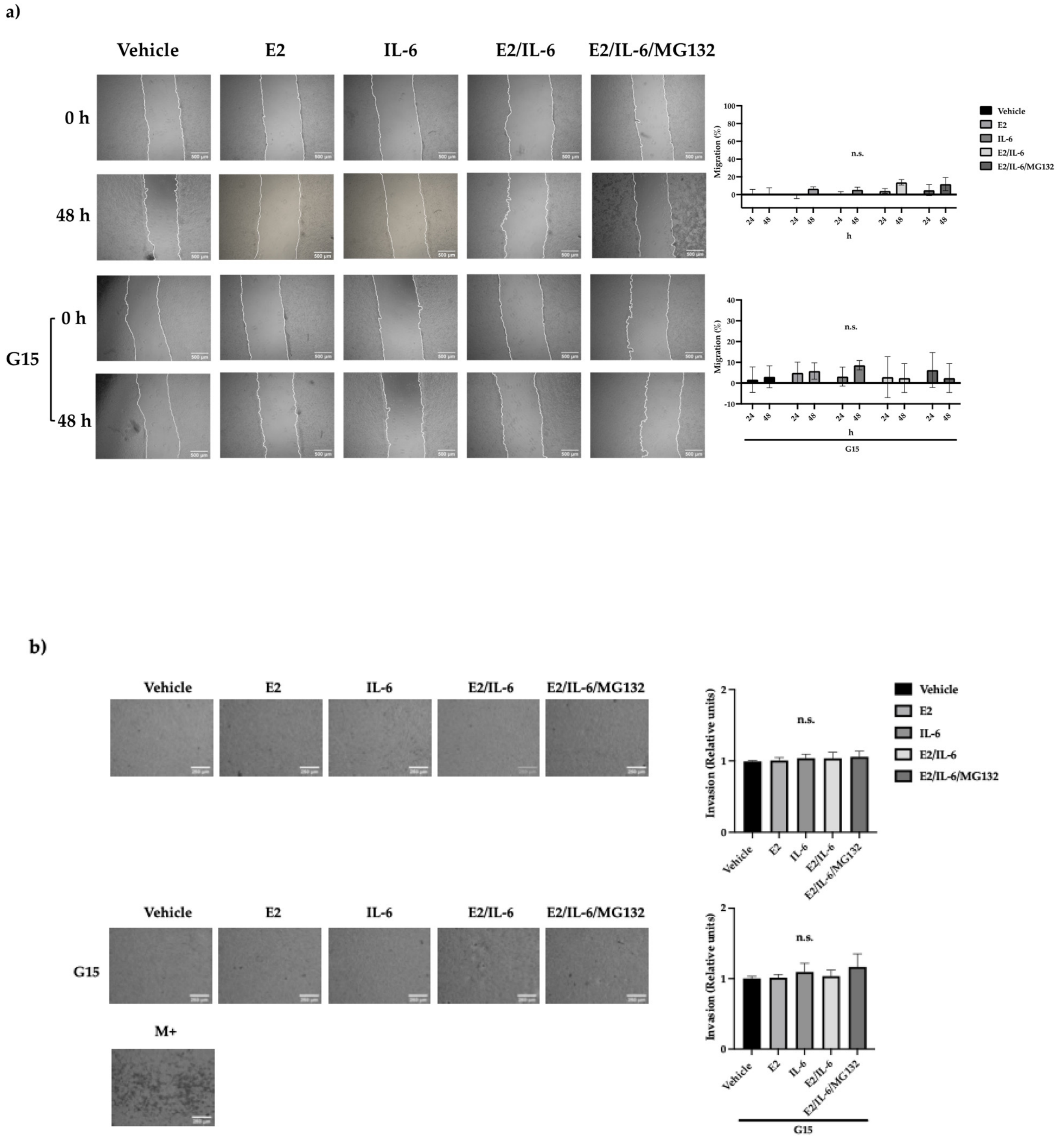

Figure S2. MCF-12 cell migration and invasion. (**a**) Cell migration was evaluated by wound healing assay at 24 and 48 h in the presence of E2 (10 nM), IL-6 (50 ng/mL), MG132 (0.03 μM), and G15 (0.625 μM). The photographs are 4X, and the scale bar represents 500 μm. Each bar shows the mean of triplicates ± SE of three independent experiments. Two-way ANOVA post hoc Tukey, $p \leq 0.05$. n.s.= not significant. (**b**) Cell invasion was assessed by

Transwell chamber assay at 24 h of stimulation with E2 (10 nM), IL-6 (50 ng/mL), MG132 (0.03 µM) and G15 (0.625 µM). M+ = Cells cultured in F-12 medium supplemented with FBS 10%. 10X photographs, scale bar represents 250 µm. Each bar shows the mean of triplicates ± SE of three independent experiments. One-way ANOVA post hoc Tukey, p≤ 0.05. n.s.= not significant.

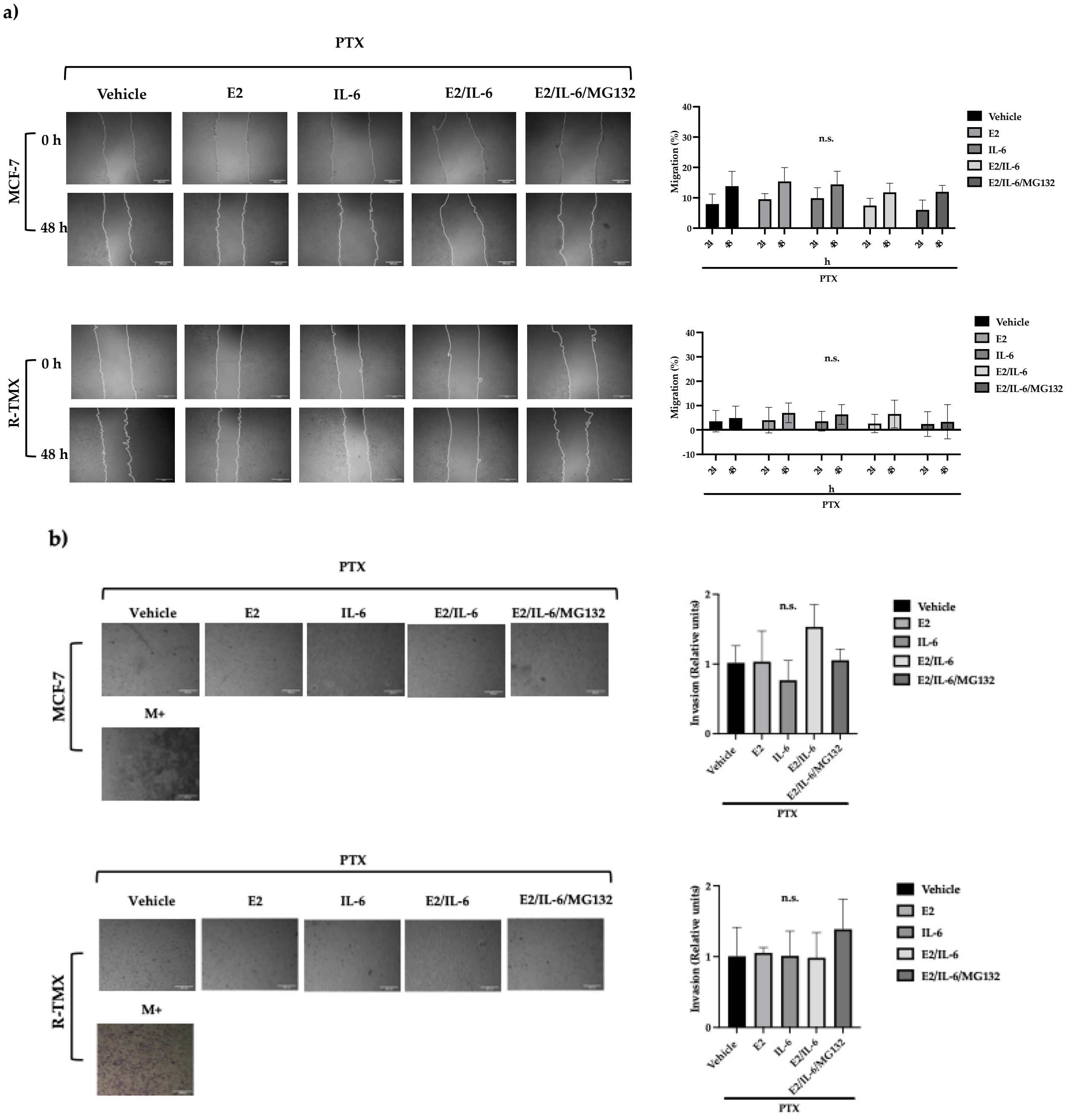

Figure S3. The PTX inhibits the MCF-7 and R-TMX cell migration and invasion. (**a**) Cell migration was evaluated by wound healing assay at 24 and 48 h in the presence of E2 (10 nM), IL-6 (50 ng/mL), MG132 (0.03 µM), and PTX (100 nM). The photographs are 4X, and the scale bar represents 500 µm. Each bar shows the mean of triplicates ± SE of three independent experiments. Two-way ANOVA post hoc Tukey, p ≤ 0.05. n.s.= not significant. (**b**) Cell invasion was assessed by Transwell chamber assay at 24 h of stimulation with E2 (10 nM), IL-6 (50 ng/mL), MG132 (0.03 µM), and PTX (100 ng/mL). M+ = Cells cultured in F-12 medium supplemented with FBS 10%. 10X photographs, scale bar represents 250 µm. Each bar shows the mean of triplicates ± SE of three independent experiments. One-way ANOVA post hoc Tukey, p≤ 0.05. n.s.= not significant.

a) b)

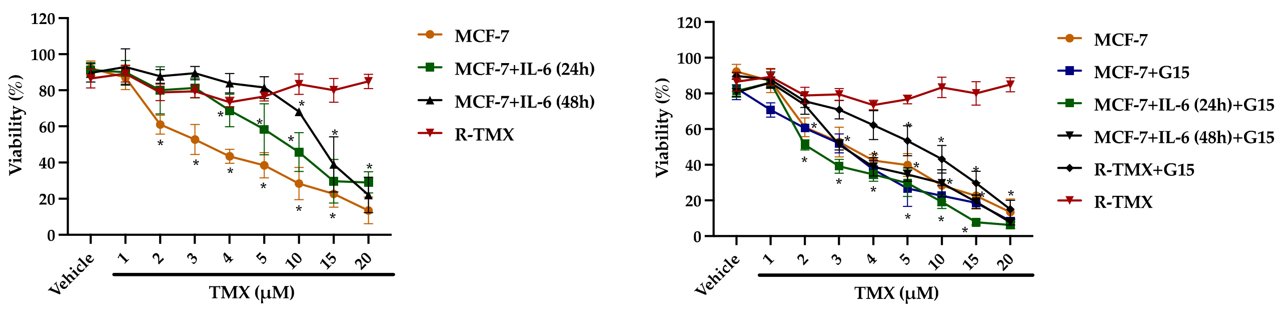

Figure S4. The GPR30 is involved in the IL-6-induced TMX resistance in luminal breast cancer. (**a**) TMX cytotoxicity was evaluated by trypan blue exclusion in unstimulated MCF-7, cells exposed to IL-6 for 24 h (IL-6 24 h) and 48 h (IL-6 48 h) and R-TMX; and (**b**) Cells were cultured as (**a**) but in the presence of G15 (0.625 µM) before exposure with TMX. Actinomycin D (16 µM) was used as a death control. The * represents statistical significance (α≤ 0.05). T-student concerning the respective vehicle of each treatment or cell line. n=3.
